# Long-Term Dietary Supplementation with Betaine Improves Growth Performance, Meat Quality and Intramuscular Fat Deposition in Growing-Finishing Pigs

**DOI:** 10.3390/foods12030494

**Published:** 2023-01-20

**Authors:** Runqi Fu, Hengzhi Zhang, Daiwen Chen, Gang Tian, Ping Zheng, Jun He, Jie Yu, Xiangbing Mao, Zhiqing Huang, Junning Pu, Wenwu Yang, Bing Yu

**Affiliations:** 1 Key Laboratory of Animal Disease-Resistance Nutrition, Ministry of Education, Ministry of Agriculture and Rural Affairs, Key Laboratory of Sichuan Province, Sichuan Agricultural University, Chengdu 611130, China; 2 Institute of Animal Nutrition, Sichuan Agricultural University, Chengdu 611130, China; 3 Skystone Feed Co., Ltd., Yixing 214258, China

**Keywords:** betaine, growing-finishing pigs, intramuscular fat, meat quality, microRNAs

## Abstract

This study was designed to investigate the effects of dietary betaine supplementation on growth performance, meat quality and muscle lipid metabolism of growing-finishing pigs. Thirty-six crossbred pigs weighing 24.68 ± 0.97 kg were randomly allotted into two treatments consisting of a basal diet supplemented with 0 or 1200 mg/kg betaine. Each treatment included six replications of three pigs per pen. Following 119 days of feeding trial, dietary betaine supplementation significantly enhanced average daily gain (ADG) (*p* < 0.05) and tended to improve average daily feed intake (ADFI) (*p* = 0.08) and decreased the feed intake to gain ratio (F/G) (*p* = 0.09) in pigs during 100~125 kg. Furthermore, a tendency to increase ADG (*p* = 0.09) and finial body weight (*p* = 0.09) of pigs over the whole period was observed in the betaine diet group. Betaine supplementation significantly increased a*_45 min_ and marbling and decreased b*_24 h_ and cooking loss in *longissimus lumborum* (*p* < 0.05), tended to increase intramuscular fat (IMF) content (*p* = 0.08), however had no significant influence on carcass characteristics (*p* > 0.05). Betaine supplementation influenced the lipid metabolism of pigs, evidenced by a lower serum concentration of low-density lipoprotein cholesterol (*p* < 0.05), an up-regulation of mRNA abundance of fatty acid synthase and acetyl-CoA carboxylase (*p* < 0.05), and a down-regulation of mRNA abundance of lipolysis-related genes, including the silent information regulators of transcription 1 (*p* = 0.08), peroxisome proliferator-activated receptorα (*p* < 0.05), peroxisome proliferator-activated receptor gamma coactivator-1α (*p* = 0.07) and carnitine palmitoyl transferase 1 (*p* < 0.05) in *longissimus lumborum*. Moreover, betaine markedly improved the expression of microRNA-181a (*miR-181a*) (*p* < 0.05) and tended to enhance *miR-370* (*p* = 0.08). Overall, betaine supplementation at 1200 mg/kg could increase the growth performance of growing-finishing pigs. Furthermore, betaine had a trend to improve meat quality and IMF content via increasing lipogenesis and down-regulating the abundance of genes associated with lipolysis, respectively, which was associated with the regulation of *miR-181a* and *miR-370* expression by betaine.

## 1. Introduction

In the past, genetic selection in pigs mainly concentrated on the needs of rapid growth, muscular development and low fat [1]. Unfortunately, this selection resulted in adverse effects on the sensory properties of pork [2]. Currently, with the increasing demand of consumers for high-quality pork, more and more researchers have concentrated on improving the meat quality characteristics of pigs [3,4]. Meat quality is comprehensively evaluated by characteristics including color, water holding capacity, tenderness and flavor, which determine the acceptability of the meat to the consumer [5]. Notably, IMF is also one of the pivotal factors in evaluating meat quality, and enhancing its content will help to facilitate muscle tenderness, flavor and juiciness [6]. The variation of IMF content in animals was influenced by many factors such as sex, genetic background, growth stage and feeding [7]. In addition, the molecular mechanism of IMF deposition, reflecting the lipogenic or lipolytic capacity of muscle, is still unclear. Nevertheless, it is a reasonable and feasible approach to improve the IMF of pigs through the nutritional strategy, which has been established in a previous study [8].

Betaine, a trimethyl derivative of the glycine [9], was first discovered in sugar beet (*Beta vulgaris*) [10], and was also presented in other food resources, such as whole grains, wheat bran and spinach [11]. Betaine is generally obtained from the two-step oxidation of choline and exogenous ingestion of daily food [12]. The main physiological function of betaine is to provide methyl and osmotic protection for the body [13]. As there are three active methyl groups in the molecular structure of betaine, it has a high transmethylation efficiency. In animal production, betaine is often used to provide methyl for animals, thereby reducing the dependence on other methyl donors, like methionine and choline [9,14]. Interestingly, Cadogan, et al. [15] observed a 14.8% reduction of backfat thickness in pigs fed with betaine-supplemented diet. Thus far, betaine is used more as a carcass conditioner in commercial feeds, which can reduce carcass fat and improve lean meat percentage of animals [16,17]. In fact, the regulation role of betaine on meat quality is still controversial. Some studies reported a decrease in carcass fat [17,18], while others indicated that there was no effect [19,20]. Several studies have also suggested that betaine was capable of increasing IMF in pigs [21,22]. In contrast, Zhong et al. [3] reported that betaine supplementation could reduce the IMF in finishing mini-pigs. Mechanistically, some current investigations proposed that betaine affected IMF content by modulating the expression of lipogenesis-related genes or the uptake of fatty acids by muscle [20,23]. 

In addition, microRNA (miRNAs) are a family of post-transcriptional gene repressors, which play important roles in animals and are involved in regulating gene expression in various metabolic systems [24]. It is generally believed that miRNAs directly bind to the 3′ untranslated region (UTR) of target genes, thus inhibiting their expression. More and more attention has been paid to understand the association between miRNAs and lipid metabolism. Additionally, previous studies have confirmed miRNAs were involved in regulating the expression of critical genes in lipid metabolism [25,26]. The association between betaine and miRNA has been focused on glucose metabolism [27], but there is little information related to lipid metabolism. Therefore, the effects of betaine on IMF with its corresponding regulatory mechanisms are inconclusive and required further investigation. Combining the above information, the purpose of this experiment was to investigate the effects of long-term betaine supplementation in growing-fattening pigs. Additionally, to mechanistically to understand whether betaine mediates lipid metabolism in pigs through the regulation of miRNA expression. The findings of this study may help us to further explore the possible mechanisms of miRNAs for IMF accumulation and contribute to clarity to the potential applications of betaine in pig production.

## 2. Materials and Methods

### 2.1. Animal and Experimental Design

Thirty-six Duroc × Landrance × Yorkshire crossbred growing pigs weighing 24.68 ± 0.97 kg were randomly assigned to two treatments (six replications and three pig per replicate). Pigs were fed a basal diet with or without betaine (1200 mg/kg). Betaine with 96% purity was supplied by Skystone Feed Co. (Jiangsu, China). The whole experiment was divided into four periods (25–50 kg, 50–75 kg, 75–100 kg and 100–125 kg) and the corresponding basal diet was formulated to meet or exceed the nutrient requirements of swine recommended by the NRC 2012 [28] (Table 1). Pigs in per replicate were fed in an individual 2.0 × 3.0 m^2^ pen, and all pigs were given *ad libitum* access to water and feed and were observed for 119 days throughout the trial.

### 2.2. Growth Performance

Feed intake of pigs per pen was recorded daily, and pigs were weighed at the beginning and end of each period. Average daily gain, average daily feed intake and the ratio of feed to gain were calculated for the four periods and the whole period.

### 2.3. Samples Collection

One pig was randomly selected from each replicate for *anterior vena cava* blood collection. The serum sample was separated by centrifugation and stored at −20 °C. Then, the selected finishing pigs were euthanized by electrocution and divided down the centerline in accordance with standard commercial procedures. Liver samples were collected for the measurement of crude fat content. The *longissimus lumborum* muscle was collected for the analysis of meat quality, IMF content and the expression of fat metabolism-related genes.

### 2.4. Analysis of Carcass Characteristics

The hot carcass of each pig was weighed individually to determine the dressing percentage. Fat thickness was determined at the first rib, the last rib and the lumbar joint of right carcass, respectively. The length between the united phalanges and the first cervical vertebra was considered the carcass length. Loin muscle area was measured at the tenth rib on the right side of carcass. The leaf fat and liver (excluding gall bladder) of each pig were weighed separately and the visceral indices were calculated based on the corresponding final body weight of the pigs.

### 2.5. Measurement of Meat Quality

Meat quality was determined with reference to a previous study [29]. Briefly, meat color (brightness, L*; redness, a*; yellowness, b*) was measured 45 min and 24 h after slaughter using a colorimeter (NR10QC, 3nh, Shenzhen, China). The pH values at 45 min and 24 h post- slaughter were determined by a calibrated pH meter (testo 205, Testo Inc., Lenzkirch, Germany). The weight change of the muscle samples before and after cooking was measured starting at 45 min after the pigs were slaughtered for calculating cooking loss. For the evaluation of instrumental tenderness, muscle samples were precooked, i.e., in a water bath heating, to bring the central temperature to 70 °C and then cooled to 4 °C. Four to six cores of 1.27 cm diameter were then removed from the treated muscle samples along the direction of muscle fibers. Shear force was quantified using Texture Analyzer (TA-XT Plus. Stable Micro Systems, Godalming, UK), accompanied by Texture Exponent software system (version 1.22, Stable Micro Systems, Godalming, UK). The blade thickness was 3 mm and the internal angle of the cutting edge was 60°. The parameters were set as follows: maximum load on the sensor was 490 N, pre-measurement speed was 1 mm/s, measurement speed was 2 mm/s, post-measurement speed was 10 mm/s, span was 40 mm and the target force was automatic. The measurement was repeated four times for each sample. Marbling of muscles was subjectively evaluated using five-points scales (one point for devoid of marbling or extremely trace distribution of marbling and five points for excessive distribution of marbling) according to the NPPC (1991) guidelines. The liver and muscle samples were pretreated by vacuum freeze-drying to remove water while recording the water content, then crushed and analyzed for fat content using FOSS Soxtec Main Unit (2055, FOSS, Hillerød, Denmark) according to the AOAC method [30].

### 2.6. Determination of Serum Hormones and Biochemical Indicators

Serum concentrations of total triglyceride and cholesterol were directly determined by commercial assay kits based on glycerol phosphate oxidase-p-aminophenazone method. According to the instruction, the low-density lipoprotein cholesterol level was determined by commercial assay kits. Enzyme linked immunosorbent assay kits was applied to quantify concentrations of very low-density lipoprotein cholesterol, growth hormone, insulin, and leptin and insulin-like growth factors-1. All kits were supplied by Nanjing Jiancheng Bioengineering Institute (Nanjing, China).

### 2.7. RNA Extraction and Real-Time RCR

Total RNA from *longissimus lumborum* muscle was extracted using Trizol reagent (TaKaRa, Dalian, China) in combination with a previous modified method [31]. RNA concentration was quantified by spectrophotometer NanoDrop (ND-2000c, Wilmington, DE, USA) together with the 260/280 ratio (between 1.8 and 2.0) to determine its purity. Agarose-formaldehyde 1.5% gels were used to detect the integrity of RNA. Then, the RNA reverse transcription product was amplified by real-time quantitative PCR (RT-PCR) on the detection system (CFX96, Bio-Rad, Hercules, CA, USA) using the SYBR Premix Ex TaqTM kit (TaKaRa, Dalian, China). All primers were commercially synthesized and technically supported by Sangon Biotech (Shanghai, China) (Tables S1 and S2). The gene sequences of mRNA and miRNA were obtained through NCBI and miRbase databases, respectively. Notably, specific stem-loop primers were applied to RT-PCR reactions for lipid metabolism-associated miRNAs, while random primers were provided for lipid metabolism-associated genes. U6 was employed as a housekeeper gene to quantify the abundance of target miRNAs. β-actin was used for standardizing the mRNA expression of genes. The RT-PCR reaction conditions were as follows: Pre-denaturation of 95 °C for 30 s, forty cycles at 95 °C for 5 s, 60 °C for 30 s and 70 °C for 60 s. The calculation of gene expression was referred to in a previous study [32].

### 2.8. Statistical Analysis

Statistical analysis was performed using SAS 9.4 (SAS Inst. Inc., Cary, NC, USA) with a two-tailed Student’s test. One replicate was used as a statistical unit for growth performance data, while an individual pig was used as the statistical unit to analyze other data. Marbling was analyzed by a chi-square test. *p* < 0.05 was considered a significant difference, and 0.05 ≤ *p* < 0.10 represented a trend.

## 3. Results

### 3.1. Growth Performance

**Figure 1:**
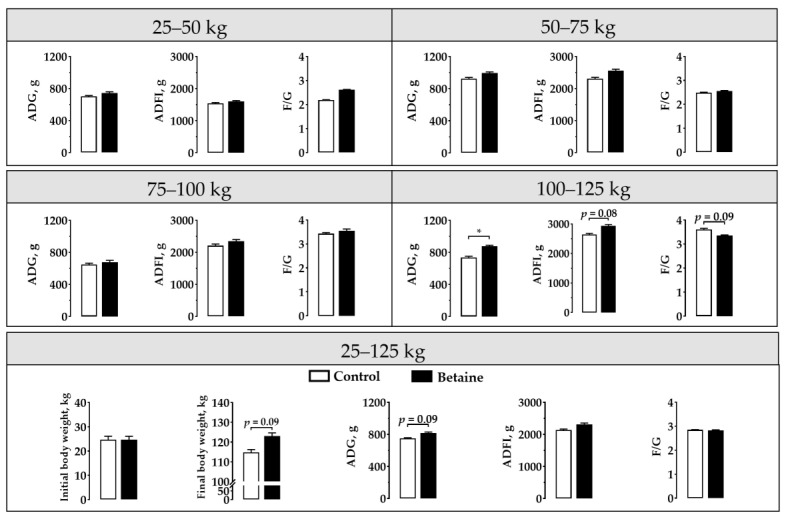
Effects of betaine on growth performance in growing-finishing pigs. ADG, average daily gain; ADFI, average daily feed intake; F/G, the feed intake to gain ratio. * *p* < 0.05 versus the control group.

As presented in Figure 1, betaine supplementation enhanced ADG (*p* < 0.05) and tended to enhance ADFI (*p* = 0.08) in growing-finishing pigs in a 100–125 kg period, while tended to decrease F/G (*p* = 0.09). Furthermore, during the whole period (25–125 kg), a tendency toward increased finial body weight (*p* = 0.09) and ADG (*p* = 0.09) was observed in pigs with supplemented betaine diets.

### 3.2. Carcass Characteristics

As shown in Table 2, compared with the control group, no significant effects on carcass characteristics were observed in pigs fed the diet with betaine (*p* > 0.05).

### 3.3. Meat Quality

As seen in Table 3, dietary betaine inclusion significantly increased a*_45 min_ and decreased b*_24 h_ in *longissimus lumborum* of growing-finishing pigs post-slaughter (*p* < 0.05). The cooking loss was decreased by dietary betaine (*p* < 0.05), whereas an elevation in marbling was observed (*p* < 0.05). Additionally, a tendency to reduce shear force was detected in pigs fed betaine diet (*p* = 0.09).

### 3.4. Visceral Index, Hepatic and Muscle Lipid Contents

**Figure 2:**
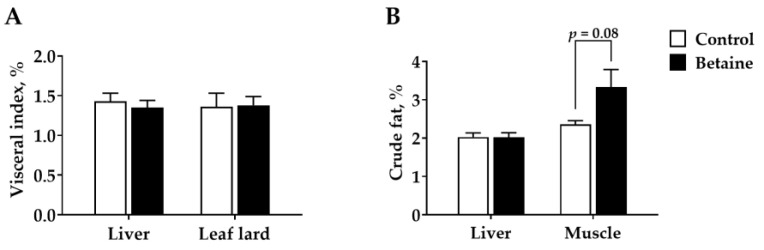
Effects of betaine on (**A**) visceral index and (**B**) hepatic lipid and intramuscular fat contents in growing-finishing pigs.

As presented in Figure 2, no significant differences were observed in the index of liver and leaf lard between betaine and control treatments (Figure 2A) (*p* > 0.05). However, there was a tendency to enhance intramuscular fat in the betaine group (Figure 2B) (*p* = 0.08).

### 3.5. Serum Hormones and Biochemical Indicators

**Figure 3:**
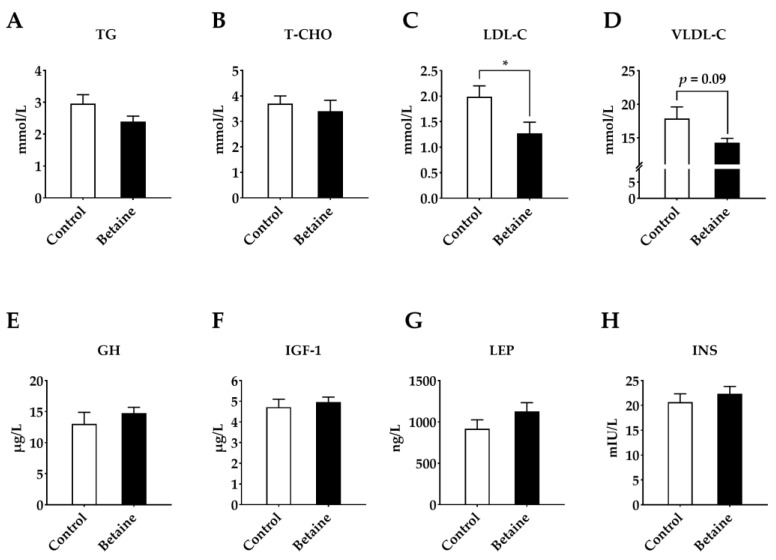
Effects of betaine on serum biochemical parameters and hormones in growing-finishing pigs. (**A**) TG, total triglycerides; (**B**) T-CHO, total cholesterol; (**C**) LDL-C, low-density lipoprotein cholesterol; (**D**) VLDL-C, very low-density lipoprotein cholesterol; (**E**) GH, growth hormone; (**F**) IGF-1, insulin-like growth factor 1; (**G**) LEP, leptin; (**H**) INS = insulin. * *p* < 0.05 versus the control group.

As seen in Figure 3, betaine supplementation decreased serum LDL-C (*p* < 0.05) (Figure 3C) and VLDL-C levels (Figure 3D) (*p* = 0.09), whereas it had no influence on GH, IGF-1, leptin and INS levels in serum (Figure 3E–H) (*p* > 0.05).

### 3.6. Expression of Lipogenic and Lipolysis Genes in Muscle

**Figure 4:**
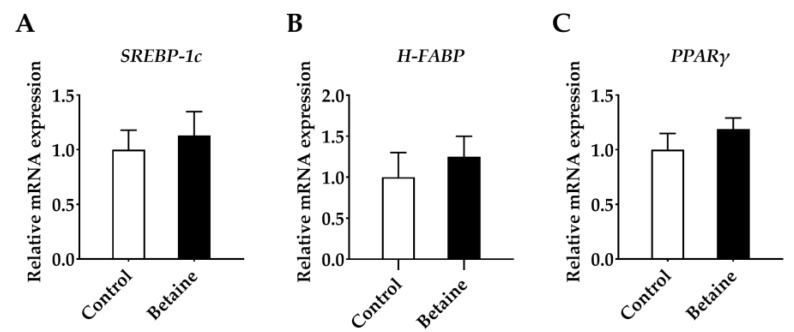

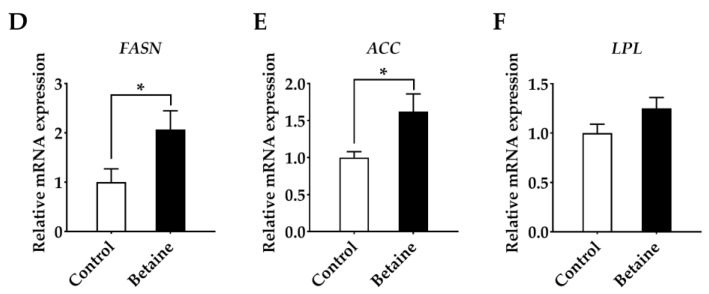
Effects of betaine on lipogenesis genes in *longissimus lumborum* muscle of growing-finishing pigs. (**A**) *SREBP-1c*, sterol regulatory element-binding protein-1c; (**B**) *H-FABP*, fatty acid-binding protein; (**C**) *PPARγ*, peroxisome proliferator-activated receptor γ; (**D**) *FASN*, fatty acid synthase; (**E**) *ACC*, acetyl-CoA carboxylase; (**F**) *LPL*, lipoprotein lipase. * *p* < 0.05 versus the control group.

**Figure 5:**
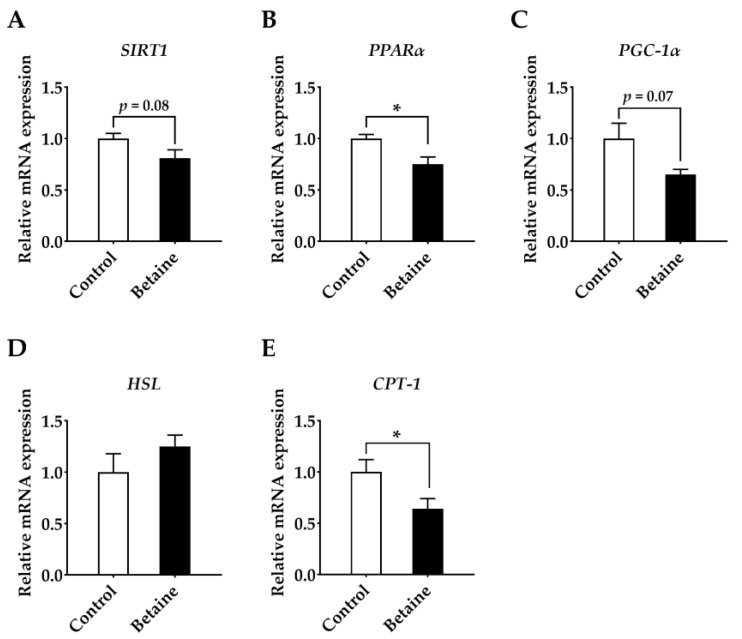
Effects of betaine on lipolysis genes in *longissimus lumborum* muscle of growing-finishing pigs. (**A**) *SIRT*, silent information regulator of transcription 1; (**B**) *PPARα*, peroxisome proliferator-activated receptorα; (**C**) *PGC-1α*, peroxisome proliferator-activated receptor gamma coactivator-1α; (**D**) *HSL*, hormone-sensitive lipase; (**E**) *CPT1*, carnitine palmitoyl transferase 1. * *p* < 0.05 versus the control group.

As displayed in Figure 4, mRNA expressions of *FASN* (Figure 4D) (*p* < 0.05) and *ACC* (Figure 4E) (*p* < 0.05) in the muscle of growing-finishing pigs were up-regulated by dietary betaine. Moreover, betaine administration clearly down-regulated the abundances of *PPARα* (Figure 5B) (*p* < 0.05) and *CPT1* (Figure 5E) (*p* < 0.05) and tended to decrease the mRNA expressions of *SIRT1* (*p* = 0.08) (Figure 5A) and *PGC-1α* (Figure 5C) (*p* = 0.07).

### 3.7. Lipid Metabolism-Related miRNAs Expression

**Figure 6:**
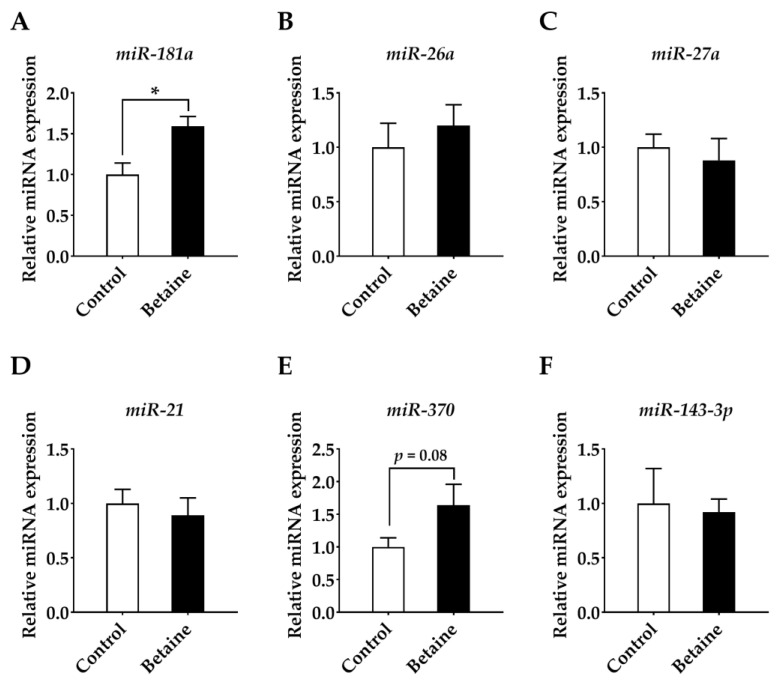
Effects of betaine on microRNA expression in *longissimus lumborum* muscle of growing-finishing pigs. (**A**) *miR-181a*; (**B**) *miR-26a*; (**C**) *miR-27a*; (**D**) *miR-21*; (**E**) *miR-370*; (**F**) *miR-143-3p*. * *p* < 0.05 versus the control group.

As some miRNAs can regulate mRNA expression, we further analyzed several miRNAs associated with lipid metabolism. As presented in Figure 6, there was a significant enhancement in the miRNA expression of *miR-181a* (Figure 6A) of muscle in pigs fed betaine diet (*p* < 0.05). Additionally, a tendency to increase the abundance of *miR-370* (Figure 6E) was discovered in pigs fed diet containing betaine (*p* = 0.08). However, pigs fed betaine diet had no impact on the expression of *miR-26a* (Figure 6B), *miR-27a* (Figure 6C), *miR-21* (Figure 6D), and *miR-143-3p* (Figure 6F) (*p* > 0.05).

## 4. Discussion

This study was designed to investigate the effects of betaine on growth performance, carcass characteristics and IMF deposition in growing-finishing pigs, and to explore the underlying mechanism. Studies on the effect of betaine (usually added at levels of 1000–1500 mg/kg) on growth performance of pigs have been reported [4,33], whereas the results were inconsistent. Specifically, Yu et al. [34] found a significant improvement of growth performance when pigs fed 1000 mg/kg betaine from 20 kg to approximately 64 kg. Furthermore, long-term dietary supplementation with betaine at 1000 mg/kg increased ADG and FBW in pigs weighing 29.6 to 100 kg [35]. While feeding 1000 mg/kg betaine did not significantly improve the growth performance of pigs (weight from 77.8 kg to 99 kg) [36]. Notably, betaine supplementation at 1250 mg/kg promoted ADG of pigs within the weight range of 40 to 60 kg [37] or 55.7–90 kg [38]. However, no influence on growth performance was observed with 1500 mg/kg betaine supplementation for pig weighing over 65 kg, while carcass composition was significantly affected by betaine [4]. Apparently, the effects of betaine on pig growth performance are probably associated with the feeding period or the dose. In this study, we found that dietary betaine supplementation at 1200 mg/kg significantly improved ADG in pigs from 100~125 kg, with a tendency to increase ADFI and decrease F/G. Furthermore, a tendency for FBW and ADG to be enhanced by betaine was observed through the whole period, and this was probably mainly attributed to the improved effect of betaine on growth performance in the last phase. Hence, these results suggested that long-term supplementation with betaine at 1200 mg/kg contributed to improve growth performance in pigs. Huang et al. [33] suggested that betaine-enhanced growth performance of pigs was associated with the increased GH secretion. Hormones play a crucial role in regulating the growth and metabolism of the body. Little information is available on the effects of betaine on hormone levels, though hormone secretion is influenced by many factors including breed, weight and stress [39]. No significant effects of betaine on serum hormone concentrations in growing-finishing pigs were revealed in this experiment.

Several studies have identified betaine as a carcass regulator involved in enhancing carcass lean and decreasing fat deposition [17,18]. Nevertheless, other experiments showed that betaine did not impact carcass traits [19,20]. It has been pointed out that the lean meat and fat content of pigs fed betaine may be affected by dietary energy level or lysine to calorie ratio [40]. Particularly, betaine exhibited a more pronounced effect on carcass characteristics when dietary energy was limited [41]. In this study, the carcass characteristics of pigs were not influenced by betaine treatment, probably due to the adequate energy and protein levels in the diet. With the increasing demand of consumers for the sensorial characteristics of meat, improving pork quality has become one of the main focuses of swine research. Meat color has a greater impact on purchasing preference than other sensory factors, because consumers regard discoloration as a primary reflection of meat freshness and quality [42]. L*, a* and b* values were utilized to quantitatively evaluate meat color. Hwang et al. [43] found that the a* value for pork improved on the first and seventh day of storage in pigs fed diets containing betaine. It is generally believed that lipid oxidation triggers the conversion of myoglobin to methemoglobin [44], causing a change in muscle color from red to an unattractive brown. Additionally, the color (b*) is used to reflect the degree of browning [45]. In this study, dietary betaine inclusion exerted a significant effect on meat color, as evidenced by an increase of a*_45 min_ and a decrease of b*_24 h_ in *longissimus lumborum*. Similar results have been reported in previous studies [17,46]. Despite there being several recognized visual sensory assessments of meat, palatability evaluation is still indispensable. Currently, our study found that betaine supplementation has a tendency to reduce the shear force, thereby improving the palatability of pork to some extent. The variability of shear force is, of course, negatively correlated with muscle fat content [5,47]. For meat industry, water-holding capacity (WHC) determines economic efficiency, so there is a considerable demand for optimizing this parameter [48]. Moreover, the WHC of pork can generally be reflected by cooking loss. While a previous study found dietary betaine had no significant effect on waster loss in *M. longissimus dorsi* and *M. semimembranosus* of Alentejano pig [22], the current trial revealed that betaine markedly reduced cooking loss in *longissimus lumborum*. The variation in WHC between different muscle tissues is reasonable and may be attributed to factors such as protein, fat content and muscle fiber type. Moisture in meat is usually negatively correlated with fat content, high-fat meat will show less water loss under the same condition [49]. These attract a great deal of interest in the effects of betaine on muscle content. Previous studies, however, have shown inconsistent results regarding the effect of betaine on muscle fat deposition in pigs. According to Martins et al. [22], the long-term addition of betaine promoted intramuscular lipid deposition, without impacting carcass fat deposition. Other studies have found an absence [50] or even a reduced effect [3] of betaine on IMF. We found that betaine significantly enhanced marbling. Additionally, there was a tendency for IMF content to increase following betaine in ingestion in pigs. Within acceptable ranges, increased IMF concentration will help to facilitate meat quality properties including juiciness, texture and flavor [51]. This is probably why the significant reduction in cooking loss and the trend towards lower shear force are influenced by the betaine in the diet.

Since lipids are the predominant taste precursors, IMF is considered to be a vital factor in response to muscle flavor [6]. Generally, genetic or nutritional approaches to improving meat quality have focused on changes in IMF [8]. It is nutritionally accepted that the excess energy is deposited into the muscle when the pig reaches its maximum rate of protein deposition, thus increasing the IMF content [52]. However, the mechanism by which dietary betaine affects IMF has not yet been determined. Albuquerque et al. [20] indicated that dietary betaine increased IMF in finishing-pigs, and the underlying molecular mechanism was mediated by lipid synthesis and fatty acid transport. Lipid metabolism is a crucial regulatory pathway in the body, and is a complex process involving the transport of lipid molecules, lipogenesis and lipolysis [53]. LDL is converted from VLDL, and is a lipoprotein particle that brings cholesterol in serum into peripheral tissue like muscle [54], promoting muscle uptake of LDL [55]. We observed that betaine decreased the concentrations of LDL-C and VLDL-C. Therefore, it was a possible speculation that most lipids were transported to peripheral tissue muscle. Muscle fat accumulation is determined by dynamic changes between lipid deposition and clearance, which is associated with the increased muscle adipogenesis and/or triglyceride export or β-oxidation. Some lipogenic and lipolytic genes play essential roles in the lipid metabolism pathway [56]. The expression of FASN and ACC, two critical enzymes for lipogenesis, was positively correlated with lipid synthesis capacity. ACC is regarded as the rate-limiting enzyme for fat production in pig muscle and has synergistic effect with FASN [57]. Our results found an up-regulation of mRNA expression of *FANS* and *ACC* in *longissimus lumborum* in pigs fed with betaine diet, suggesting that the modulatory effect of betaine on IMF may be due to the increase of lipogenesis. Fat content is not only controlled by the rate of de novo lipid production in a specific tissue, but also by the rate of lipolysis [52]. Therefore, another potential presumption for elevated IMF content in *longissimus lumborum* of betaine-fed pigs could be the reduced rate of muscle lipolysis. CPT-1 is the rate-limiting enzyme in lipolytic metabolism, participated in the hydrolysis of triglycerides [56]. In comparison to lean pigs, obese types had more fat reserves, and showed lower mRNA abundance, enzyme activity or protein expression of CPT-1 [58]. Lipolysis can thereby be attenuated by reducing the expression of CPT-1. In addition, SIRT1 is a regulator of cellular metabolism, with functions in modulating transcription and metabolism. Activated SIRT1 induces high expression of CPT-1 and low expression of FASN and ACC in muscle tissue [59]. PPARα is identified as a principal transcription factor-regulated gene engaged in fatty acid oxidation. High expression of PGC1α in skeletal muscle could stimulate mitochondrial biogenesis and induce more fatty acid oxidation [60]. In our study, the mRNA expression levels of *CPT-1*, *SIRT1*, *PPARα* and *PGC1α* were decreased in betaine when compared to control groups, suggesting that the regulation of betaine on lipid catabolism was also non-negligible.

At present, the IMF-enhancing effect of betaine may be the co-regulatory result of lipogenesis and lipolysis. However, studies on the mechanism of betaine on lipid metabolism in pigs have mainly focused on the expression of genes related to lipid metabolism [20,21], while there is little information about miRNAs-related data. The relationship between betaine and miRNAs has been mentioned in previous studies [27,61]. Here, we further explored whether the lipid metabolism regulation of betaine was mediated by miRNA. miRNAs are a family of post-transcriptional gene suppressors that widely exist in animals and are strongly correlated with the modulation of gene expression under various conditions, encompassing almost all aspects of systemic regulation of metabolism [24]. miRNAs have been suggested to be capable of controlling the expression of critical genes in lipid metabolism [26]. Earlier studies have shown that miR-181a repressed SIRT1 expression by directly binding to the 3′ UTR [25]. The present study revealed that betaine supplementation increased the *miR-181a* abundance and simultaneously decreased the mRNA expression of *SIRT1*, indicating that the influence of betaine on increasing IMF content by reducing *SIRT1* expression may be regulated by miR-181a. Furthermore, although betaine significantly reduced the mRNA expression level of *PPARα*, there was no significant alteration in its corresponding *miR-21* [62]. Thus, the effect of betaine on *PPARα* expression may not be mediated by *miR-21*. Previous work identified CPT1 as a target of miR-370 [26]. We observed an increased expression levels of *miR-370* and a decreased mRNA abundance of *CPT1* in pigs fed betaine diet. While the levels of *miR-27a* and *PPARγ* expression were not affected by betaine, which appeared to emphasize the importance of *miR-181a* and *miRNA-370* for the regulation of lipolytic metabolism of IMF. These results demonstrated that betaine affected the expression of *SIRT1* and *CPT1* via *miR-181a* and *miR-370*, respectively, thereby mediating the lipid metabolism of IMF. However, it is still unclear whether betaine affects IMF content by directly or indirectly regulating miRNAs expression. More studies are needed to further confirm whether the potential mechanism of betaine regulating IMF metabolism is mediated by lipid metabolism-related miRNAs.

## 5. Conclusions

In conclusion, long-term supplementation of 1200 mg/kg of betaine in the diet improved the growth performance of growing-finishing pigs, and was beneficial to meat quality, especially with a tendency to increase IMF content. Betaine increased lipogenesis and moderated lipolytic reactions in muscle, thus contributing to the improvement of IMF. Notably, *miR-181a* and *miR-370* may be involved in the modulation of crucial genes in IMF lipid metabolism. The present study provides a molecular basis for explaining the potential mechanisms by which betaine improves IMF deposition in growing-finishing pigs.

## Figures and Tables

**Table 1:** Composition and nutrients levels of basal diet (air dry basis, %).

Items	Phase			
	25–50 kg	50–75 kg	75–100 kg	100–125 kg
Ingredients, %				
Corn	76.55	80.30	84.00	88.74
Soybean meal	16.71	15.43	10.87	6.20
Wheat bran	0.00	0.00	1.00	1.20
Fish meal	2.70	0.00	0.00	0.00
Soybean oil	1.40	1.40	1.50	1.40
Limestone	0.73	0.70	0.63	0.59
Dicalcium phosphate	0.47	0.66	0.53	0.43
L-Lysine·HCL	0.49	0.51	0.48	0.46
DL-Methionine	0.08	0.07	0.07	0.06
L-Threonine	0.15	0.16	0.15	0.15
L-Tryptophan	0.04	0.04	0.04	0.04
NaCl	0.30	0.35	0.35	0.35
Chloride choline	0.15	0.15	0.15	0.15
Vitamin premix ^1^	0.03	0.03	0.03	0.03
Mineral premix ^2^	0.20	0.20	0.20	0.20
Total	100.00	100.00	100.00	100.00
Calculated values				
Digestible energy, MJ/kg	14.23	14.23	14.23	14.23
Moisture, %	12.31	11.88	11.73	11.71
Crude protein, %	15.69	13.75	12.13	10.44
Crude fat, %	4.48	4.48	4.66	4.63
Crude fiber, %	2.22	2.19	2.05	1.87
Calcium	0.66	0.59	0.52	0.46
Available phosphorus	0.31	0.27	0.24	0.21
Lysine, %	1.03	0.90	0.78	0.66
Methionine, %	0.30	0.25	0.23	0.20
Threonine, %	0.62	0.55	0.49	0.43
Tryptophan, %	0.18	0.16	0.14	0.12

^1^ provided the following per kg of diets: Vitamin A, 9000 IU; Vitamin D3, 3000 IU; Vitamin E, 20 IU; Vitamin K3, 3.0 mg; Vitamin B1, 1.5 mg; Vitamin B2, 4.0 mg; Vitamin B6, 3.0 mg; Vitamin B12, 0.2 mg; Niacin, 30 mg; Pantothenic, 15 mg; Folic acid, 0.75 mg; Biotin, 0.1 mg. ^2^ provided the following per kg of diets, 25–50 kg: Fe (FeSO_4_·H_2_O) 60 mg, Cu (CuSO_4_·5H_2_O) 4 mg, Mn (MnSO_4_·H_2_O) 2 mg, Zn (ZnSO_4_·H_2_O) 60 mg, I (KI) 0.14 mg, Se (Na_2_SeO_3_) 0.2 mg; 50–75 kg: Fe 50 mg, Cu 3.5 mg, Mn 2 mg, Zn 50 mg, I 0.14 mg, Se 0.15 mg; 75–125 kg: Fe 40 mg, Cu 3 mg, Mn 2 mg, Zn 50 mg, I 0.14 mg, Se 0.15 mg.

**Table 2:** Effects of betaine on the carcass characteristics in growing-finishing pigs.

Items	Control	Betaine	SEM	*p*-Value
Dressing percentage, %	74.49	74.62	0.57	0.86
Carcass length, cm	103.33	104.67	1.68	0.57
Backfat thickness, cm	1.79	1.84	0.14	0.80
Loin muscle area, cm^2^	45.82	48.14	3.50	0.64

**Table 3:** Effects of betaine on meat quality in growing-finishing pigs.

Items	Control	Betaine	SEM	*p*-Value
Color parameters				
L*_45 min_ (lightness)	44.03	43.25	0.55	0.34
a*_45 min_ (redness)	4.04	5.11	0.34	<0.05
b*_45 min_ (yellowness)	2.85	2.82	0.32	0.92
L*_24 h_ (lightness)	53.84	53.00	1.00	0.54
a*_24 h_ (redness)	6.45	6.62	0.38	0.74
b*_24 h_ (yellowness)	4.26	3.52	0.28	<0.05
pH_45 min_	6.36	6.31	0.16	0.70
pH_24 h_	5.49	5.53	0.07	0.57
Cooking loss, %	36.35	32.82	0.98	<0.05
Shear force, N ^1^	45.18	38.20	2.06	0.09
Marbling ^2^	1.42	2.30	-	<0.05

Note: ^1^ N, Newtons. ^2^ Marbling scale (5-point scales), where 1 = devoid of marbling or extremely trace distribution of marbling and 5 = excessive distribution of marbling.

## Data Availability

Data is contained within the article and Supplementary Materials.

## Supplementary Materials

The following supporting information can be downloaded at: https://www.mdpi.com/article/10.3390/foods12030494/s1, Table S1: Primer sequences used for quantitative real-time PCR; Table S2: Primer sequences of miRNAs and U6 used for real-time PCR.
