# Supplementary material for: Long-Term Dietary Supplementation with Betaine Improves Growth Performance, Meat Quality and Intramuscular Fat Deposition in Growing-Finishing Pigs
Source: Foods. 2023 Jan 20;12(3):494. doi: 10.3390/foods12030494 (PMC9914383; doi:10.3390/foods12030494)
Supplement: Supplementary file 1 [file foods-12-00494-s001.zip › foods-2107361-supplementary corrected.pdf]

foods

MDPI

Supplementary Material

Table S1. Primer sequences used for quantitative real-time PCR.

Gene	Froward	Reverse	Product length(bp)	Accession number
ACTB	5'-TCTGGCACCACACCTTCT-3'	5'-TGATCTGGGTCATCTTCTCAC-3'	114	XM_021086047.1
SREBP-1c	5'-GCGACGGTGCCTCTGGTAGT-3'	5'-CGCAAGACGGCGGATTTA-3'	218	XM_021066226.1
H-FABP	5'-ATGACCAAGCCTACCACA -3'	5'-AGTTTGCCTCCATCCAGT-3'	170	NM_001099931.1
FASN	5'-AGCCTAACTCCTCGCTGCAAT-3'	5'-TCCTTGGAACCGTCTGTGTTC-3'	196	NM_001099930.1
ACC	5'-AGCAAGGTCGAGACCGAAAG-3'	5'-TAAGACCACCGGCGGATAGA-3'	169	XM_021066238.1
LPL	5'-CACATTCACCAGAGGGTC-3'	5'-TCATGGGAGCACTTCACG-3'	177	XM_021072174.1
PPARγ	5'-CCAGCATTTCCACTCCACACTA-3'	5'-GACACAGGCTCCACTTTGATG-3'	124	XM_005669788.3
SIRT1	5'-TTGATCTTCTCATTGTTATTGGGTC-3'	5'-ACTTGGAATTAGTGCTACTGGTCTTA-3'	62	NM_001145750.2
PPARα	5'-CGACCTGGAAAGCCCGTTAT-3'	5'-GAGGCTTTGTCCCCACAGAT-3'	279	NM_001044526.1
PGC-1α	5'-GATGTGTCGCCTTCTTGTTC -3'	5'-CATCCTTTGGGGTCTTTGAG-3'	93	XM_021100444.1
CPT-1	5'-GACAAGTCCTTCACCCTCATCGC-3'	5'-GGGTTTGGTTTGCCCAGACAG-3'	170	XM_021091195.1
HSL	5'-GCCTTTCCTGCAGACCATCT-3'	5'-CACTGGTGAAGAGGGAGCTG-3'	104	NM_214315.3

Table S2. Primer sequences of miRNAs and U6 used for real-time PCR

miRNA	Primer sequences	Accession number
miR-27a	F: CGGCGGTTCACAGTGGCTAAG<br>RT: GTCGTATCCAGTGCAGGGTCCGAGGTATTCGCACTGGATACGACGCGGAA	MIMAT0002148
miR-181a	F: CGGCGAACATTCAACGCTGTCGG<br>RT: GTCGTATCCAGTGCAGGGTCCGAGGTATTCGCACTGGATACGACAACTCA	MIMAT0010191
miR-143-3p	F: CGCTGAGTTGAGATGAAGCACTG<br>RT: GTCGTATCCAGTGCAGGGTCCGAGGTATTCGCACTGGATACGACGAGCTA	MIMAT0013879
miR-370	F: CCGGCCTGCTGGGGTGGA<br>RT: GTCGTATCCAGTGCAGGGTCCGAGGTATTCGCACTGGATACGACACCAGG	MIMAT0025373
miR-122	F: CGCGGTGGAGTGTGACAATGG<br>RT: GTCGTATCCAGTGCAGGGTCCGAGGTATTCGCACTGGATACGACACAAAC	MIMAT0002119
miR-26a	F: CGCGCGCGTTCAAGTAATCCAGGA<br>RT: GTCGTATCCAGTGCAGGGTCCGAGGTATTCGCACTGGATACGACAGCCTA	MIMAT0002135
miR-21	F: CGGCGGTAGCTTATCAGACTGA<br>RT: GTCGTATCCAGTGCAGGGTCCGAGGTATTCGCACTGGATACGACTCAACA	MIMAT0002165
Universal reverse primer	R: ATCCAGTGCAGGGTCCGAGG	
U6	F: CTCGCTTCGGCAGCACA<br>R: AACGCTTCACGAATTTGCGT<br>RT: AACGCTTCACGAATTTGCGT	
